# Diagnostic performance of serum pepsinogen assay for the prediction of atrophic gastritis and gastric neoplasms: Protocol for a systematic review and meta-analysis

**DOI:** 10.1097/MD.0000000000014240

**Published:** 2019-01-25

**Authors:** Chang Seok Bang, Jae Jun Lee, Gwang Ho Baik

**Affiliations:** a Department of Internal Medicine; b Institute of New Frontier Research; c Department of Anesthesiology and Pain Medicine, Hallym University College of Medicine, Chuncheon, Korea.

**Keywords:** atrophic, gastric neoplasms, gastritis, pepsinogens

## Abstract

**Background:**

Serum pepsinogen assay (sPGA) combining concentration of pepsinogen I (PG I), and the ratio of PG I/II is the noninvasive biomarker for predicting chronic atrophic gastritis (CAG) and neoplasms reflecting mucosal secretory status. Although various cut-off values have been suggested, PG I ≤70 ng/mL and PG I/II ≤3 have been widely accepted. However, previous studies for diagnostic test accuracy presented only pooled outcomes, which cannot discriminate the diagnostic validity of sPGA with cut-off of PG I ≤70 ng/mL and PG I/II ≤3.

**Methods:**

We will search the core databases [MEDLINE (through PubMed), the Cochrane Library, and Embase] from their inception to December 2018 by 2 independent evaluators. The P.I.C.O. is as follows; Patients: who have histologically proven CAG or gastric neoplasms, Intervention: sPGA with cut-off of PG I ≤70 ng/mL and/or PG I/II ≤3, Comparison: none, Outcome: diagnostic performance indices of sPGA for CAG and gastric neoplasms (sensitivity, specificity, positive predictive value, negative predictive value, likelihood ratios) (if, true/false positive, true/false negative values are presented, diagnostic performance indices will be calculated). All types of study design with full text will be sought and included. The risk of bias will be assessed using the QUADAS-2 tool. Descriptive data synthesis is planned and quantitative synthesis (bivariate and HSROC model) will be used if the included studies are sufficiently homogenous. Publication bias will be assessed.

**Results:**

The results will provide clinical evidence for diagnostic validity of sPGA.

**Conclusion:**

This study will provide evidence of sPGA for predicting CAG and gastric neoplasms.

## 1 Introduction

Gastric cancer is a global health-related burden and the fourth most common cause of cancer-related deaths worldwide.^[1]^ The histopathologic cascade for the development of intestinal-type gastric adenocarcinoma is from normal gastric epithelium to chronic gastritis, chronic atrophic gastritis (CAG), and intestinal metaplasia (IM), followed by dysplasia, and gastric cancer in sequence.^[2]^ Patients with premalignant lesions, such as an AG or dysplasia also have a considerable risk of developing gastric cancer and early detection of these lesions are important for the screening of gastric cancer.^[3]^

For the population-based screening of gastric cancer, endoscopic mass screening programs have shown its efficacy where gastric cancer prevalent countries such as Korea and Japan.^[4]^ Endoscopic screening program has reduced gastric cancer-related mortality by 47% in a nested-case control study in Korea.^[5]^ However, it is not cost-effective in regions with low incidence of gastric cancer and stepwise- or individualized screening according to the risk factors of gastric cancer has been recommended.^[4]^

Except for the endoscopic diagnosis using visual inspection (with or without image-enhanced endoscopy) or histologic diagnosis using such as an updated Sydney system for CAG or IM, serum pepsinogen assay (sPGA) combining concentration of pepsinogen I (PG I), and the ratio of PG I/II has been the noninvasive biomarker for predicting CAG and neoplasms reflecting gastric mucosal secretory status.^[6]^ Although various cut-off values have been suggested, PG I ≤70 ng/mL and PG I/II ≤3 have been widely accepted for the prediction of CAG or gastric cancer.^[7,8]^ However, previous study for diagnostic test accuracy (DTA) presented only pooled outcomes, which cannot discriminate the diagnostic validity of sPGA with cut-off of PG I ≤70 ng/mL and PG I/II ≤3.^[9]^ Another studies of DTA showed combined test accuracy of sPGA with *Helicobacter pylori (H pylori)* antibody^[10]^ and/or gastrin-17,^[11,12]^ for the prediction of gastric cancer^[10]^ and CAG,^[11,12]^ which cannot discriminate the diagnostic validity of sPGA. This study aimed to provide evidence of sPGA for predicting CAG and gastric neoplasms.

## 2 Methods

This systematic review and meta-analysis will fully adhere to the principles of the Preferred Reporting Items for Systematic reviews and Meta-Analyses (PRISMA-P) checklist.^[13]^ This study protocol was registered at PROSPERO (https://www.crd.york.ac.uk/prospero) on December 2018 (registration number, CRD42018116470) before study was initiated. The approval of institutional review board was exempted due to the characteristics of this study (collecting and synthesizing data from published studies).

### 2.1 Literature searching strategy

MEDLINE (through PubMed), the Cochrane library, and Embase will be searched using common keywords relevant to sPGA, CAG, and gastric neoplasms (from inception to December 2018) by 2 independent evaluators (CSB and JJL). Medical Subject Heading or Emtree keywords will be selected for searching electronic databases. The abstracts of all identified studies will be reviewed to exclude irrelevant publications. Full-text reviews will be performed to determine whether the inclusion criteria are satisfied in the remaining studies, and the bibliographies of relevant articles will be rigorously reviewed to identify additional studies. Disagreements between the evaluators will be resolved by discussion or consultation with a third evaluator (GHB). We made searching strategy that maximizes sensitivity because searching too specifically has a risk of missing relevant literature. The detailed searching strategy is described in Table 1.

**Table 1:**
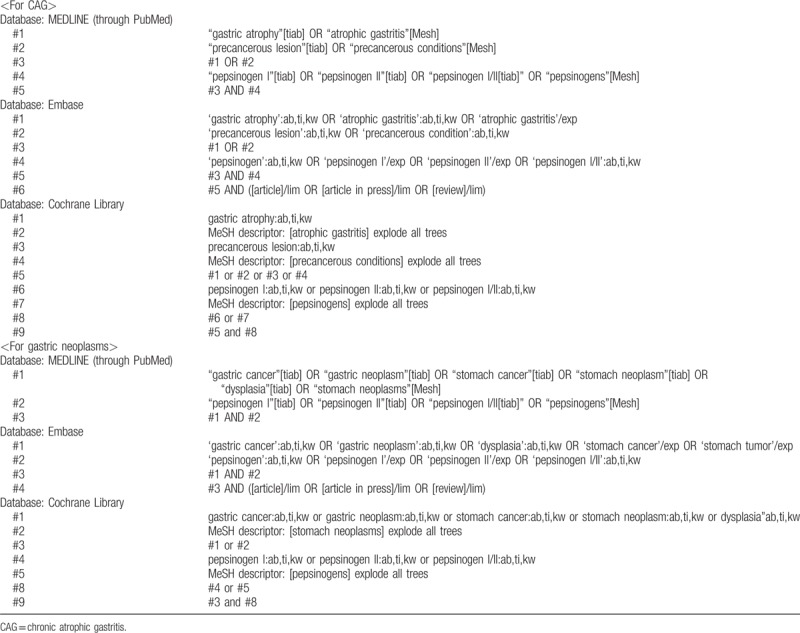
Searching strategy to find the relevant articles.

### 2.2 Selection criteria

We will include studies that met the following criteria: patients: who have histologically proven atrophic gastritis or gastric neoplasms; intervention: sPGA with cut-off of PG I ≤70 ng/mL and/or PG I/II ≤3; comparison: none; 4. outcome: diagnostic performance indices of sPGA for CAG and gastric neoplasms (sensitivity, specificity, positive predictive value, negative predictive value, and likelihood ratios) (if, true/false positive, true/false negative values are presented, diagnostic performance indices will be calculated); study design: all types (case–control studies will be analyzed by subgroup because it can exaggerate the performance of diagnostic accuracy because of selection bias); studies of human subjects; and full-text publications. Studies that met all of the inclusion criteria will be sought and selected. The exclusion criteria are as follows: review articles; guidelines, consensus documents or expert position papers; comments, letters, brief reports, proceedings, or protocol studies; publications with incomplete data; and meta-analysis articles. Studies meeting at least 1 of the exclusion criteria will be excluded from this analysis. The language of publication will not be restricted.

### 2.3 Methodological quality

The methodological quality of the included publications will be assessed using the Quality Assessment of Diagnostic Accuracy Studies-2 (QUADAS-2) tool.^[14]^ The QUADAS-2 tool contains 4 domains, including “patient selection,” “index test,” “reference standard,” and “flow and timing” (flow of patients through the study and timing of the index tests and reference standard).^[14]^ The methodological quality assessment process consists of 4 phases; report the signaling review question; develop review-specific (-tailoring) guidance; review the published flow diagram for the primary study; judgement on risk of bias and concerns about applicability.^[14]^ Each domain is determined to exhibit high-, low-, or unclear risk of bias, and the first 3 domains are also determined to exhibit high-, low-, or unclear concerns about applicability.^[14]^ The results of methodological quality assessment will be described using tabular presentation for each study and graphical presentation for overall assessment. Two of the evaluators (CSB and JJL) will independently assess the methodological quality of all the included studies, and any disagreements between the evaluators will be resolved by discussion or consultation with a third evaluator (GHB.).

### 2.4 Data extraction and primary and modifier-based analyses

Two evaluators (CSB and JJL) will independently use the same data fill-in form to collect the primary summary outcome and modifiers in each study, and disagreements between the 2 evaluators will be resolved by discussion or consultation with a third author (GHB).

DTA is the primary outcome of this study. We will calculate the diagnostic performance indices of sPGA for CAG and gastric neoplasms (sensitivity, specificity, positive predictive value, negative predictive value, likelihood ratios) using 2 × 2 tables whenever possible from the original articles that contain the number of cases for true positive (TP) (subjects with positive sPGA who have histologically proven CAG or gastric neoplasms), false positive (FP) (subjects with positive sPGA who do not have histologically proven CAG or gastric neoplasms), true negative (TN) (subjects with negative sPGA who do not have histologically proven CAG or gastric neoplasms), and false negative (FN) (subjects with negative sPGA who have histologically proven CAG or gastric neoplasms). If only a part of data is presented, we will calculate the DTA using the following formulas; Sensitivity: TP/(TP+FN); Specificity: TN/(FP+TN); Positive predictive value: TP/(TP+FP); Negative predictive value: TN/(FN+TN); Positive likelihood ratio: sensitivity/(1-specificity); Negative likelihood ratio: (1-sensitivity)/specificity; Accuracy: (TP+TN)/(TP+FP+FN+TN); Diagnostic odds ratio (DOR): (TP × TN)/(FP × FN); Standard error: (ln (upper confidence interval) – ln(lower confidence interval))/3.92 = √ (1/TP+1/FP+1/FN+1/TN).

The following data will also be extracted from each study, whenever possible; study design, age or ethnicity of enrolled population, sample size, published year, diagnostic method of index test [how many biopsy specimens were obtained and where was the site of taking biopsy specimen (body, antrum, or both) to make a diagnosis of CAG or gastric neoplasms] and technical specification of sPGA.

Narrative (descriptive) synthesis is planned and quantitative synthesis [bivariate random model^[15]^ and hierarchical summary receiver operating characteristic (HSROC) model^[16]^] will be used if the included studies are sufficiently homogenous. The common effect size will be extracted or calculated from each study and pooled meta-analysis of crude outcomes of each study with summary outcomes will be presented (i.e., paired forest plot of pooled sensitivity or specificity with confidence region and prediction region using bivariate model). SROC curve will be generated and presented using HSROC model. Heterogeneity across the studies will be determined by correlation coefficient between logit transformed sensitivity and specificity by bivariate model^[15]^ and asymmetry parameter, β (beta), where β = 0 corresponds to a symmetric ROC curve in which the DOR does not vary along the curve by HSROC model.^[16,17]^ We will also perform subgroup analyses and meta regression using the modifiers identified during the systematic review to confirm the robustness of the main result and to identify the reason of heterogeneity.

### 2.5 Statistical analysis

Stata Statistical Software, version 13.0 (College Station, TX)^[18]^ will be used for this meta-analysis (relevant packages for analyses; metandi, midas, and mylabels). Paired forest plot of pooled sensitivity or specificity with confidence region and prediction region and SROC curve will be presented. Heterogeneity across the studies will be determined by correlation coefficient between logit transformed sensitivity and specificity and asymmetry parameter, β of SROC curve. We will also perform subgroup analyses and meta-regression using the modifiers. Publication bias will be evaluated using funnel plot.

## 3 Discussion

This is the protocol of a systematic review and meta-analysis for the diagnostic performance of sPGA for the prediction of CAG and gastric neoplasms. PG I and PG II are proenzymes of pepsin, an endoproteinase of gastric juice.^[19]^ PG I is secreted mainly by chief cells in the fundic glands of the fundus and body, whereas PG II is secreted by the all gastric glands and the proximal duodenal mucosa (Brunner's glands).^[4,19–21]^ The gastric mucosal secreting ability is usually intact in noninfected or acutely infected state of *H pylori*.^[21]^ However, when chronic *H pylori* infection with CAG progresses extending from antrum to corpus of stomach, chief cells are replaced by pyloric glands.^[6]^ Therefore, concentration of serum PG I decreases as the secreting ability of gastric mucosa is damaged, whereas the concentration of PG II is relatively intact, leading to a low PG I/II ratio and this value reflects the severity of CAG.^[6,21]^ Various cut-off values have been suggested; however, PG I ≤70 ng/mL and PG I/II ≤3 have been widely accepted for the prediction of CAG or gastric cancer.^[7,8]^

SPGA combining the serum concentration of PG I and the ratio of PG I/II has been the noninvasive biomarker for predicting CAG and neoplasms reflecting mucosal secretory status.^[6]^ However, previous meta-analysis presented only pooled outcomes, which cannot discriminate the diagnostic validity of sPGA with cut-off of PG I ≤70 ng/mL and PG I/II ≤3.^[9]^ Serum concentration of gastrin, which is produced and secreted primarily by G cells in the antrum is increased when the corpus mucosa is predominantly involved, and decreased with antral predominant gastric atrophy.^[4,21]^ Although combined efficacy of sPGA, with *H pylori* antibody^[10]^ and/or gastrin-17,^[11,12]^ for the prediction of gastric cancer^[10]^ and CAG^[11,12]^, has been suggested and mainly used in Europe (as panel test), sPGA has been preferred to the serum gastrin measurement because of better reflecting gastric mucosal status.^[21]^ Moreover, previous meta-analyses could not discriminate the diagnostic validity of sPGA alone.^[10–12]^ Although one previous meta-analysis reported diagnostic validity of sPGA with cut-off of PG I ≤70 ng/mL and PG I/II ≤3 as a manner of subgroup analysis, this study was published in 2004 and recently published data could not be reflected.^[8]^

The results of this study will provide clinical evidence of diagnostic validity of sPGA for predicting CAG and gastric neoplasms.

## Acknowledgment

Funding for this research was provided by the Bio & Medical Technology Development Program of the National Research Foundation (NRF) and by the Korean government, Ministry of Science and ICT (MSIT) (grant number NRF2017M3A9E8033253).

## Author contributions

**Conceptualization:** Chang Seok Bang.

**Data curation:** Chang Seok Bang, Jae Jun Lee, Gwang Ho Baik.

**Formal analysis:** Chang Seok Bang, Jae Jun Lee

**Funding acquisition:** Chang Seok Bang.

**Investigation:** Chang Seok Bang, Jae Jun Lee, Gwang Ho Baik.

**Methodology:** Chang Seok Bang.

**Project administration:** Chang Seok Bang.

**Resources:** Chang Seok Bang, Jae Jun Lee, Gwang Ho Baik.

**Supervision:** Chang Seok Bang.

**Visualization:** Chang Seok Bang

**Writing – original draft:** Chang Seok Bang.

**Writing – review & editing:** Chang Seok Bang.

Chang Seok Bang orcid: 0000-0003-4908-5431.
